# A Composite Chitosan-Reinforced Scaffold Fails to Provide Osteochondral Regeneration

**DOI:** 10.3390/ijms20092227

**Published:** 2019-05-07

**Authors:** Alice Roffi, Elizaveta Kon, Francesco Perdisa, Milena Fini, Alessandro Di Martino, Annapaola Parrilli, Francesca Salamanna, Monica Sandri, Maria Sartori, Simone Sprio, Anna Tampieri, Maurilio Marcacci, Giuseppe Filardo

**Affiliations:** 1 Applied and Translational Research (ATR) Center, IRCCS—Istituto Ortopedico Rizzoli, 40136 Bologna, Italy; a.roffi@biomec.ior.it (A.R.); ortho@gfilardo.com (G.F.); 2 Knee Joint Reconstruction Center—3rd Orthopedic Division, Humanitas Clinical Institute, 20089 Rozzano, Italy; elizaveta.kon@humanitas.it (E.K.); maurilio.marcacci@humanitas.it (M.M.); 3 Department of Biomedical Sciences, Humanitas University, Rozzano, 20090 Milan, Italy; 4 Hip and Knee Replacement Department, IRCCS—Istituto Ortopedico Rizzoli, 40136 Bologna, Italy; 5 Laboratory of Preclinical and Surgical Studies, IRCCS—Istituto Ortopedico Rizzoli, 40136 Bologna, Italy; milena.fini@ior.it (M.F.); annapaola.parrilli@ior.it (A.P.); francesca.salamanna@ior.it (F.S.); maria.sartori@ior.it (M.S.); 6 II Orthopedic and Traumatologic Clinic; IRCCS Istituto Ortopedico Rizzoli, 40136 Bologna, Italy; alessandro.dimartino@ior.it; 7 Institute of Science and Technology for Ceramics, National Research Council (ISTEC-CNR), 48018 Faenza, Italy; monica.sandri@istec.cnr.it (M.S.); simone.sprio@istec.cnr.it (S.S.); anna.tampieri@istec.cnr.it (A.T.)

**Keywords:** chitosan, osteochondral, bone, cartilage, failure, scaffold

## Abstract

Several biomaterials have recently been developed to address the challenge of osteochondral regeneration. Among these, chitosan holds promises both for cartilage and bone healing. The aim of this in vivo study was to evaluate the regeneration potential of a novel hybrid magnesium-doped hydroxyapatite (MgHA), collagen, chitosan-based scaffold, which was tested in a sheep model to ascertain its osteochondral regenerative potential, and in a rabbit model to further evaluate its ability to regenerate bone tissue. Macroscopic, microtomography, histology, histomorphometry, and immunohistochemical analysis were performed. In the sheep model, all analyses did not show significant differences compared to untreated defects (*p* > 0.05), with no evidence of cartilage and subchondral bone regeneration. In the rabbit model, this bone scaffold provided less ability to enhance tissue healing compared with a commercial bone scaffold. Moreover, persistence of scaffold material and absence of integration with connective tissue around the scaffolds were observed. These results raised some concerns about the osteochondral use of this chitosan composite scaffold, especially for the bone layer. Further studies are needed to explore the best formulation of chitosan-reinforced composites for osteochondral treatment.

## 1. Introduction

Chondral and osteochondral defects are challenging problems, since the native structure of cartilage and subchondral bone cannot be regenerated with any of the available treatments [1,2]. The most recent strategy for treating these conditions is based on the development of new biomaterials, potentially able to induce regeneration of both cartilage and subchondral bone, preferably through a one-step procedure in order to reduce costs and morbidity for the patient, and to avoid the hurdles of cells manipulation regulation [3].

Several biomaterials have been investigated as potential substrates for osteochondral repair [4,5], and some of these have also been introduced into clinical use, although with controversial results. Whereas a porous bilayer poly(lactic-*co*-glycolic acid) (PLGA)–calcium–sulfate construct showed poor quality of repair tissue [6,7], a more promising option consisted of a biphasic type I collagen-hydroxyapatite (Coll/HA) nanophase biomimetic scaffold obtained by bio-inspired mineralization [8]. However, positive clinical results obtained even in challenging conditions, such as complex lesions [9] or unicompartmental osteoarthritis [10], were coupled with significant bone layer signal abnormalities detected at magnetic resonance imaging (MRI) [11,12]. In fact, subchondral bone is currently emerging as the main challenge for the achievement of optimal osteochondral regeneration [13,14]. Even though these findings did not correlate with worse clinical outcomes, and despite a slow but significant improvement of regenerating tissue scores, this aspect underlines the limits of this biomaterial potential to restore damaged articular tissue, at cartilage but even more at bone level.

Chitosan is attracting the interest of biologists and material scientists, due to its good biocompatibility and reabsorption property; moreover, it can be combined with collagen by intermolecular interactions, to create scaffolds with enhanced mechanical strength [15,16,17]. Thus, its use has been widely investigated for cartilage and bone healing [18,19,20], and it has also been introduced into clinical practice for the treatment of cartilage lesions, with promising results [21]. On the other hand, evidence of its potential to address osteochondral defects is still limited.

Therefore, in the present study a hybrid osteochondral cell-free scaffold, obtained by a blending process, to achieve a collagen and chitosan (col+chit)-reinforced blend, and by a bio-inspired mineralization process, to grow MgHA nanoparticles on col+chit blends (MgHA/(col+chit)), was developed and tested in vivo. A pilot study was performed on a sheep model to ascertain the potential for the treatment of critical size osteochondral defects. Furthermore, due to the key role of subchondral bone for the success of osteochondral treatment, a rabbit model was used to evaluate bone isolated from the articular environment, and its contribution toward the overall osteochondral regeneration induced by this chitosan-reinforced scaffold.

## 2. Results

### 2.1. Rabbits

#### 2.1.1. Post-Implant and Macroscopic Evaluations

Anesthesia procedures and postoperative healing period were uneventful for all animals. When femoral condyles were retrieved, a macroscopic analysis revealed some fibrotic peri-articular tissues in one case for the control group and in five cases for the experimental group. Three of the 12 samples, both in the control and in the experimental group, were not included in the evaluation because the scaffolds were not fully surrounded by trabecular bone, not allowing a fair assessment and comparison with fully trabecular bone-surrounded implants.

#### 2.1.2. Microtomography

**Figure 1:**
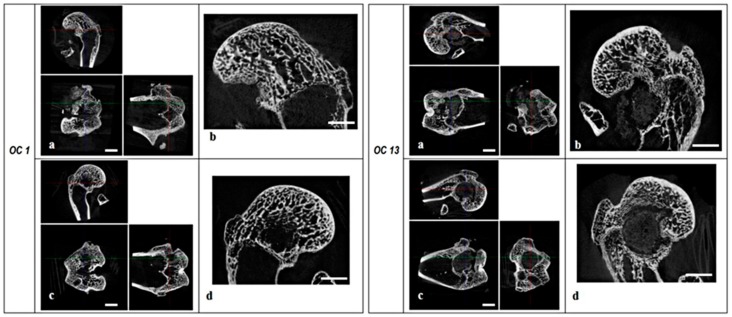
RegenOss (**left panel**) and experimental group (**right panel**) micro-CT sections. Organization of each panel: (**a**) Micro-CT sections of the right leg samples in the axial, sagittal, and coronal planes, scale bar 5 mm; (**b**) Lateral condyle sagittal micro-CT section (**right leg samples**), scale bar 3 mm; (**c**) micro-CT sections of the left leg samples in the axial, sagittal, and coronal planes, scale bar 5 mm; (**d**) lateral condyle sagittal micro-CT section (**left leg samples**), scale bar 3 mm.

Limited growth of new bone inside the defect and a thin trabecular new bone structure were detected in the control group (Figure 1), whereas no integration was observed in the experimental group where a clear separation between bone and scaffold could be detected (Figure 1). 3D morphometric analysis showed, overall, less new bone formation inside the defect (BV/TV_1_) in the experimental group (See Supplementary Materials), with lower Tb.Th inside the defect and higher Tb.Sp compared to control (both *p* < 0.05). Finally, no intergroup differences were observed on peri-implant bone 3D morphometric parameters.

#### 2.1.3. Histology and Histomorphometry

**Figure 2:**
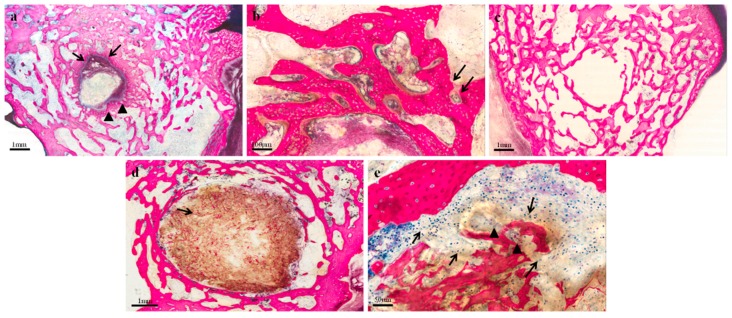
Histological images of control (**a,b,c**) and experimental groups (**d,e**). Stevenel’s blue and Gieson Pichrofucsin Staining. (**a**) residuals of material (arrow) surrounded by a continuous connective tissue (arrow heads). Magnification 2×. (**b**) Woven bone matrix containing numerous densely packed and spherical osteocytes entrapped within new trabeculae, and osteoid matrix deposited by osteoblast lining new trabeculae (arrow). Magnification 20×. (**c**) Bone tissue in progressive growth from the host bone to the area originally occupied by the scaffold. Magnification 2×. (**d**) Presence of material inside the defect area (arrow) Magnification 4×. (**e**) An inflammatory infiltrate was present inside the fibrous connective tissue around the material; this was mainly characterized by the presence of polymorphonucleated cells (arrows) and macrophages (arrow heads). Magnification 40×.

Residuals of scaffold material were described in three cases in the control group. They were surrounded by connective tissue (Figure 2a) with an intense bone activity, characterized by woven bone matrix containing numerous densely packed and spherical osteocytes entrapped within new trabeculae, and the osteoid matrix deposited by osteoblast lining new trabeculae (Figure 2b). In six cases, the scaffold was reabsorbed and bone tissue progressively grew from the host bone into the implantation area (Figure 2c).

Conversely, the scaffold material MgHA/(col+chit) was always recognizable in the bone defect area in the experimental group. No direct contact between bone tissue and scaffold was observed in seven cases (Figure 2d), due to interposed connective tissue characterized, in six of the seven specimens, by the presence of an inflammatory cell infiltrate (Figure 2e).

Finally, statistical analysis revealed no significant differences between experimental scaffold and control material regarding dynamic histomorphometric parameters (MAR and BFR, *p* = 0.138 and *p* = 0.176, respectively.

### 2.2. Sheep

#### 2.2.1. Post-Implant and Macroscopic Evaluations

All animals well tolerated surgery and survived the post-surgical period. No gait abnormalities due to sever limp or instabilities were observed. No signs of inflammation or adhesions were detected after joint analysis.

Macroscopic evaluation of untreated group samples revealed no bone or cartilage defect healing in one case, and incomplete bone defect filling and irregularity of the bone–cartilage surface in the remaining ones. In the MgHA–collagen–chitosan group, irregularity of the bone–cartilage surface and no bone defect healing occurred in all cases, with connective tissue surrounding the material. In addition, voluminous cysts in the bony compartment were present in two cases.

No significant intergroup differences were reported using the modified Fortier score [22], while the Niederaurer score [23] showed better results for untreated defects compared to treated ones, when located at the medial femoral condyle (*p* < 0.05) (Table 1).

#### 2.2.2. Microtomography

**Figure 3:**
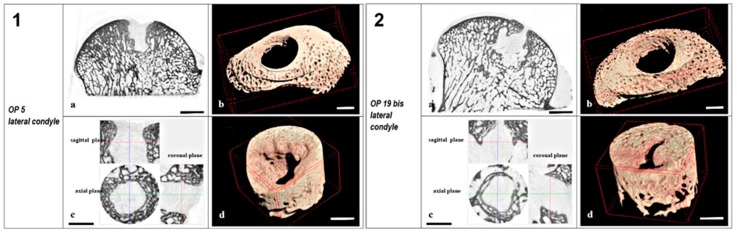
Micro-CT sections and 3D models of samples of control (**1**) and experimental group (**2**) in sheep. Each panel showed: (**a**) Condyle sagittal Micro-CT section, scale bar 5 mm; (**b**) 3D model of peri-implant trabecular bone in TV2, scale bar 3 mm; (**c**) micro-CT sections in axial, sagittal, and coronal planes of TV1, scale bar 3 mm; and (**d**) 3D model of the new bone formed into TV1, scale bar 3 mm.

**Figure 4:**
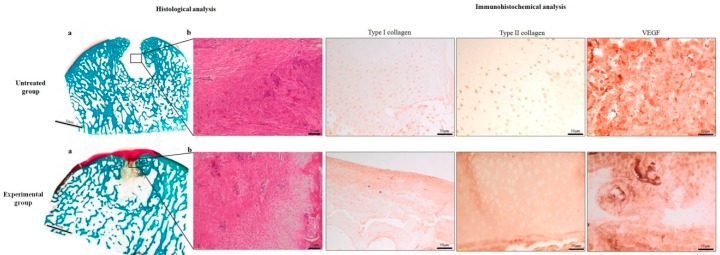
Histological evaluations at six months in the untreated group: (**a**) No evidence of bone and cartilage defect healing, Safranin-O/Fast green stain. Magnification 1×. (**b**) Presence of fibrous tissue in the defect area. H/H staining: Magnification 20×. In the experimental group: (**a**) Safranin-O/Fast green stain. Magnification 1×. (**b**) Cartilage defect filled with fibrous tissue resembling fibrocartilage. H/H staining: Magnification 20×. The immunohistochemical panel showed the staining for type I, type II collagen, and VEGF. Magnification 10×.

In the untreated group, incomplete new subchondral bone formation was detected in the defect (Figure 3) characterized by the presence of bone cysts and a concave surface. In the MgHA–collagen–chitosan group, the new bone growth was not uniformly present and large cystic areas were observed around the residual material (Figure 4), even above the 5 mm depth of the considered VOI (TV_1_) (Supplementary Materials). 

3D morphometric analysis on bone inside the defect and around the implant did not show significant differences between groups with a large overall variability. Moreover, the 2D distribution of bone inside and surrounding the defect was analyzed by sections and did not show any significant difference between groups.

#### 2.2.3. Histology 

Histology assessment of the control group confirmed the macroscopic results, showing amorphous fibrous tissue filling the defect in one sample, and an incomplete defect filling with irregular bone–cartilage surface in the other cases. Further analysis revealed an altered chondrocyte distribution, grouped in clusters, in comparison with native cartilage tissue.

In six samples of the experimental group, fibrocartilage tissue was filling the defect site, with intense proteoglycan red staining with Safranin-O (Figure 4). In the remaining two samples, no cartilage healing was detected. All samples showed no subchondral bone healing, with cysts within trabecular tissue delimited by a thick connective membrane. Comparable results were confirmed also by the semi-quantitative analysis performed with the Pineda score (Table 2).

#### 2.2.4. Immunohistochemistry

Both the untreated and MgHA–collagen–chitosan groups revealed type I and II collagen staining either negative or positive in scattered areas (Figure 4). A comparable positive staining in the cartilage extra-cellular matrix (ECM) was observed at VEGF immunohistochemical analysis, extended throughout the repair tissue up to the joint space.

## 3. Discussion

The main finding of this study is that the hybrid MgHA–collagen–chitosan scaffolds failed to provide both bone and cartilage regeneration in vivo.

The regenerative potential of this scaffold was tested in two animal models: A pilot study on a sheep model was performed to investigate the osteochondral regenerative potential, and a rabbit model was used to investigate the specific contribute of the bone layer to the overall osteochondral regeneration provided by this scaffold. The two models contributed to demonstrate the lack of regenerative potential at both the bone and cartilage level of this MgHA–collagen–chitosan scaffold. The rabbit model had previously been shown to be suitable for studying the effectiveness of different biomaterials on bone repair and regrowth [24], with an extra-articular access to bone, which allowed avoidance of evaluation bias due to the possible influence of the joint environment to bone formation. In addition, a sheep model was used as a more clinically-relevant benchmark, in order to investigate scaffold-assisted osteochondral regeneration. In fact, limb-loading between sheep and humans is comparable and the relevant loading conditions allows one to adopt a surgical technique similar to the one used in humans. Accordingly, it is generally agreed that results obtained in large animals are more representative of the clinical situation [25,26].

The pilot evaluation in sheep clearly provided evidence for a lack of regenerative potential, thus advising against the translation of this hybrid MgHA–collagen–chitosan osteochondral scaffold into clinical use. These negative results are in contrast with other reported applications of chitosan in humans. In fact, a chitosan-based gel (BST-CarGel^®^, Smith & Nephew, Andover, MA, USA) has been successfully applied in the clinical practice to treat chondral defects, after preclinical findings in an ovine model showing increased volume and hyaline-like repair tissue, with increased glycosaminoglycans (GAG) and collagen content, compared to microfracture controls [19]. After anecdotal evidence showing it to be safe and after good preliminary results [27], a multicenter randomized controlled trial highlighted greater lesion filling and superior repair tissue quality at MRI in the BST-CarGel^®^ group compared with microfracture treatment alone at 12 months [28]. In addition, histological assessment performed at one-year in a subset of patients confirmed the improvement of tissue quality seen at MRI [29]. Stable clinical findings and MRI superiority were later confirmed at five years of follow-up [21]. Recently this approach was also applied to hip chondral defects [30].

An important aspect that may explain the difference between the good findings documented in clinical practice and the poor results of this hybrid MgHA–collagen–chitosan-based scaffold is the type of lesion treated. While only chondral defects were previously addressed in literature, our experiments involved osteochondral lesions, where bone was also considered as a target. The scaffold failed to provide bone regeneration in the two investigated models: The scaffold material and the bone defect area were always recognizable in both models and presented large cystic formations surrounding the residual material. The problems related to bone formation potential were confirmed by the comparison, specifically in the bone targeted rabbit model, of this material with a commercially available product for bone regeneration. Therefore, the subchondral component of the biphasic implant may have jeopardized the overall tissue quality of the treated osteochondral unit.

Chitosan was deemed to be a good material for bone regeneration in several previous preclinical studies. Gupta et al. [31] showed that a chitosan–agarose–gelatin scaffold produced both hyaline-like cartilage and bone regeneration, well integrated within the surrounding tissues in rabbit osteochondral defects. Deng et al. [32] implanted a silk fibroin–chitosan scaffold in the same animal model, observing partial scaffold degradation and partial osteochondral regeneration. These were further enhanced by bone marrow-derived mesenchymal stem cells (MSCs), suggesting that the scaffold can act as a carrier for MSCs, with a possible application in tissue engineering strategies. Beside these positive findings, the use of chitosan as regenerative material for the regeneration of the articular surface presents challenges, as highlighted by the results of the present study.

ECM components play a critical role in regulating the expression of the chondrocyte phenotype. Given the importance in stimulating chondrogenesis, the use of GAG or GAG analogues as components of a cartilage tissue scaffold, appears to be a logical approach. Chitosan is a chitin derivative, partially de-acetylated and found in arthropod exoskeletons. It shares some structural characteristics with various GAGs and hyaluronic acid also present in articular cartilage; therefore, it represents a promising candidate for chondral and osteochondral regeneration [33]. Multiple studies have been published investigating the properties of chitosan, such as biocompatibility, biodegradability, etc. [34,35,36], with the ability to be processed into porous structures being one of the most promising features for cell transplantation and tissue regeneration purposes [37]. Furthermore, chitosan–nanoHA composites have demonstrated to be rapidly biodegradable, nontoxic, and prone to chemical and enzymatic modification, with a structure similar to GAGs found in bone ECM, thus stimulating cell adhesion, proliferation, and osteoinduction [38]. However, besides an overall evidence for chitosan-based biomaterials to provide both cartilage and bone regeneration, significant variations in material properties can be obtained, depending on the source and preparation procedure, and this can heavily impact on the biological response [39]. To this regard, the importance of chitosan properties for several tissue engineering applications has been previously reported [40]. Abarrategi et al. [4] confirmed these findings also for osteochondral defects, showing a considerable influence of different material properties on in vivo tissue regeneration on the same rabbit model applied in this study, depending on chitosan mineral content, molecular weight, and deacetylation degree.

Therefore, heterogeneous results may be obtained with different chitosan formulations, and further complexity is due to the need of blending different biomaterials with chitosan in order to improve its properties, as it lacks proper mechanical strength for bone tissue engineering and its interface does not promote easy cell adhesion [41]. The scaffold tested in this study was a composite based on a new innovative concept of bio-inspired mineralization of self-assembling collagen fibers with nano-apatites (MgHA) presenting bio-competent ion substitutions (e.g., Mg^2+^, CO_3_^2−^) [42,43]. Bio-inspired mineralization is a low cost, versatile process that can, in principle, be applied to various bio-polymers to generate composite macromolecular matrices with enhanced, tailored properties [44]. It also enables the development of fibrous constructs with graded mineralization, thus reproducing the different compartments of osteochondral tissues by a compositional and morphological perspective [8]. Moreover, the generated 3D fibrous hybrid bone scaffolds demonstrated good osteogenic and regenerative ability in vivo [45]. Based on this, the hybrid MgHA–collagen–chitosan scaffold seemed a promising option for osteochondral regeneration. Nonetheless, our research failed to provide evidence for any benefit in both medium-size and big-size animal models.

Even though fibrotic peri-articular tissue and some inflammatory cells infiltrates were found, no foreign body reaction or adverse immunological rejection was documented. No significant differences were shown on peri-implant bone 3D morphometric parameters between the experimental group and control. However, direct contact between implant and bone tissue was absent in most of the samples, which could explain the less abundant formation of new bone inside the defect in the experimental group. These microtomography findings were also confirmed by histology and histomorphometry analyses. While in the control group the scaffold was reabsorbed and bone tissue progressively grew from the host bone to the area originally occupied by the scaffold, in the experimental group the scaffold material was always recognizable together with the bone defect area, with scarce or absent contact at the interface. Thus, the lack of bone integration potential of this new biomaterial may have impaired the overall regenerative properties of the osteochondral scaffold as well. To this regard, it has been underlined that the molecular weight of chitosan is inversely proportional to the hydration capacity of the chitosan itself, being a greater molecular weight correlated to lower swelling [46]. For this reason, the biomaterial for scaffold development was selected based on low molecular weight. However, the high cross-linking degree, performed to control the morphological and chemical stability of the scaffold, probably reduced the availability of active charged sites involved in water adsorption on the chitosan surface, thus hampering the deposition of proteins relevant for focal adhesion of osteoprogenitor cells, promoting the formation of fibrous tissue surrounding the scaffold and preventing cell colonization.

Moreover, the crosslinking treatment performed on the material with the aim of improving the scaffold stability in in vivo conditions, may have induced a too high cross-linking degree of the matrix, with a strong impact on its biodegradability properties. This aspect is coherent with in vivo studies reported by Abarrategi et al. [4], showing that a scaffold with a too low biodegradation can compromise tissue regeneration activating, in some cases, negative processes.

Therefore, our results raise some concerns on the osteochondral application of chitosan, particularly regarding the subchondral bone layer, which is essential in supporting the overlying chondral regeneration. While chitosan seems to be a promising option for osteochondral regeneration thanks to its safety, low cost, and overall positive literature findings, the choice of the starting material, as well as trimming material properties and interaction with other materials, deserve careful consideration. In fact, beside the biological rational and some promising findings, this study further underlines the importance for any biomaterial formulation to be tested in preclinical animal models for each specific tissue application, in order to provide empirical data and explore its potential as well as its limitations in vivo, before osteochondral regeneration can be targeted in the clinical practice.

## 4. Materials and Methods

### 4.1. Scaffold Preparation

#### 4.1.1. Bone Scaffold Preparation

A 70:30 wt.% ratio collagen–chitosan blend was prepared and subjected to bio-inspired mineralization to obtain a hybrid magnesium-doped HA/(collagen and chitosan) composite (MgHA/(col+chit)), in the shape of a 3D scaffold [8].

Two hundred and forty-four mL of H_3_PO_4_ (Sigma Aldrich, Milan, Italy, purity ≥ 85 wt.%) solution (0.040 M) were added to the collagen and chitosan (70/30 wt.%) blend. This acidic gel was then dropped into a basic suspension obtained dispersing 1.20 g of Ca(OH)_2_ (Sigma Aldrich, Milan, Italy, purity ≥ 95 wt.%) in 200 mL of distilled water supplemented with Mg^2+^ ions (MgCl_2_ 6H_2_O, Sigma Aldrich, purity ≥ 99 wt.%) to yield a composite material MgHA/(col+chit) with a theoretical ratio of 70:30 wt.% and with the aspect of a cream-colored gel. This process was carried out to induce the heterogeneous nucleation of apatite nano-nuclei presenting magnesium substitution.

The MgHA/(col+chit) gel was centrifuged until a dense gel was obtained, in order to achieve a 3D low-porous and hard construct, which was then poured into multi-well plates (6 mm diameter, 8 mm height) and freeze-dried (5 Pascal, Cinquepascal srl, Milan, Italy) at −40°C for complete water elimination and pore formation, in order to achieve a highly porous structure to allow high cell penetration and migration from the subchondral bone-like layer to the cartilaginous one. Only the bone layer was used for the medium-size animal study.

#### 4.1.2. Cartilaginous Layer Scaffold Preparation

Low molecular weight chitosan (purchased from Sigma Aldrich, Milan, Italy) was dissolved in a 0.5 M acetic acid solution to obtain a 1 wt.% chitosan concentration; 21.4 g of this solution was slowly mixed with 50 g of 1 wt.% type I collagen (OPOCRIN Spa, Formigine, Italy), suspended in acetic buffer (final pH = 3.5). This suspension was then precipitated through a cofibration process by adding NaOH 0.5 M to increase the pH from 3.5 to 5.5. The formation of small fibrous aggregates of 70:30 wt.% col+chit blend was observed. The homogeneous precipitate was then washed three times with 300 mL of water, centrifuged, and freeze-dried.

#### 4.1.3. Osteochondral Scaffold Preparation

Both materials were treated with the cross-linking agent 1,4-butanediol diglycidyl ether (BDDGE, (purchased from Sigma Aldrich, Milan, Italy)) through immersion for 48 h (24 h at room temperature followed by 24 h at 5 °C) in a BDDGE aqueous solution (2.5 mM), thus setting up a BDDGE:collagen ratio equal to 1 wt.%; they were then washed three times to remove any residual that did not react.

Both gels, mineralized and not mineralized, were spread on a Mylar^®^ sheet (polyester film, anti-adhesion treated; by Dupont Tejin Films, Hopewell, VA, USA) producing thin uniform layers that were interpenetrated to obtain monolithic bi-layer constructs and then freeze-dried (MgHA–collagen–chitosan). Finally, the monolithic scaffolds were gamma-sterilized at 25 Kgray before in vivo implantation.

### 4.2. Surgical Procedures

In vivo studies were performed complying with the European and Italian Law on animal experiments, after the approval (N.0044454 date 27/12/2012 and N.0044451 date27/12/2012) of the research protocols by the Ethical Committee of Rizzoli Orthopedic Institute and by the responsible public authorities in agreement with EU regulations (EU Directive 2010/63/EU for animal experiments). All animals were purchased from authorized farms and submitted to a quarantine period before their utilization.

#### 4.2.1. Rabbit Model

Twelve adult male New Zealand rabbits (Harlan Laboratories SRL, S. Pietro al Natisone, Udine), were housed in standard and controlled conditions (22 ± 1 °C, 55% ± 5% RH) and fed with standard diet (Mucedola, Milano, Italy) and water ad libitum.

Animals underwent surgery under general anesthesia. The lateral distal femoral epiphysis was exposed through a 2 cm longitudinal skin incision and cylindrical critical size defects (diameter: 6 mm, length: 8 mm) were created bilaterally by using a drill. Scaffolds were implanted according to the group allocation: Group 1 received the RegenOss scaffold (commercially available control) and group 2 the MgHA/(col+chit) scaffold. Soft tissues were then sutured and routine antibiotics and analgesics were administered postoperatively. After 8 weeks, animals were euthanized with i.v. administration of Tanax^®^ (Hoechst AG, Frankfurt am Main, Germany), under anesthesia. Femoral epiphyses were then processed for histological, histomorphometric and microtomographic evaluations.

#### 4.2.2. Sheep Model

Six crossbred adult sheep, 65 ± 5 kg b.w. (Breeder: Pancaldi Raffaele, Bologna, Italy), were housed in single boxes in standard and controlled conditions (22 °C ± 1 °C, 50% ± 5% RH, ventilation of 10 air changes per hour) and fed with standard pellet diet (Mucedola, Milan, Italy) and water ad libitum. Animals were divided into 2 groups: Group 1 untreated (*n* = 2) and group 2 treated with the osteochondral MgHA–collagen–chitosan scaffold (*n* = 4). Surgery was conducted under general anesthesia with the animals in dorsal recumbence. The right medial and lateral condyles were exposed through a median skin incision and parapatellar joint approaches. A specifically designed drill was used to create a cylindrical osteochondral defect (7 mm diameter, 5 mm depth) in the weight-bearing area of both condyles. In group 2, grafts were implanted with a press-fit technique, while in the control group defects were left empty. Routine antibiotics and analgesics were administered postoperatively. All animals were euthanized 6 months after surgery by Tanax^®^ (Intervet-Italia S.r.l., Milan, Italy) injection under general anesthesia. Stifle joints were carefully opened and macroscopically evaluated for signs of inflammation, such as tissue reddening, hypertrophy of the villous part of the synovial membrane, tissue adhesions, appearance of a fat pad, and clearness and color of the synovial fluid. The macroscopic appearance of the implants was blindly assessed using the modified Fortier [22] and the Niederauer scoring systems [23].

Finally, each medial and lateral sample was cut in half along the central axis of the implant and evaluated through microtomography, histology, and immunohistochemistry.

### 4.3. Explant Evaluation

#### 4.3.1. Microtomography

High resolution microtomography Skyscan 1176 was used for sample scans (Bruker Micro-CT, Kontich, Belgium) at a voltage of 50 kV, current of 500 μA, and with an aluminum filter of 0.5 mm.

Each sample was rotated by 180° with a rotation step of 0.4° according to the following set up: Rabbit, average frame of 2, nominal resolution 9 μm; sheep, average frame of 3, nominal resolution 17.5 μm. The acquired images were reconstructed by the NRecon software (1.6.8.0) with corrections for alignment beam hardening and ring artefact reduction, and 3D models were created to allow visualizations of the samples in space.

Two specific volumes of interest (VOIs) were defined in each sample. TV_1_: cylindrical VOI with diameter of 6 mm and height of 8 mm or diameter of 7 mm and height 5 mm for rabbits and sheep, respectively. These were then used to evaluate the material and the new bone formation into the defect. TV_2_: VOI of the trabecular bone of the condyle in the area around the defect with the same height of TV_1_ (used to evaluate peri-implant bone).

#### 4.3.2. Morphological Parameters

1. Defect BV/TV_1_ (%), ratio between the volume of newly formed bone within the bone defect and the total volume of the bone defect;
2. Defect Trabecular Thickness Tb.Th (mm), calculated in a model-independent way [47] over the total volume of the bone defect TV_1_;
3. Defect Trabecular Number Tb.N (mm^−1^), the number of intersections through a trabecular structure per unit length of a random linear path through TV_1_;
4. Defect Trabecular Separation Tb.Sp (mm), calculated as the Tb.Th;
5. Peri-implant BV/TV_2_ (%), ratio between the volume of trabecular bone around the bone defect and the total volume of interest TV_2_;
6. Peri-implant Tb.Th Trabecular Thickness, Tb.N Trabecular Number and Tb.Sp Trabecular Separation calculated as above (in TV_2_) (see Supplementary Materials).

#### 4.3.3. Histology and Histomorphometry

Rabbits: Femoral distal epiphyses were processed without prior decalcification. After fixation and dehydration, samples were embedded in poly-methylmetacrilate (Merck, Schuchardt, Hohenbrunn, Germany), then sectioned perpendicularly to the bone surface into sections (100 ± 10 μm thick) that were then grounded to a thickness of 15 ± 5 µm (Saphir apparatus 550, ATM GmbH, Mammelzen, Germany). Three sections for each sample were left unstained for oxytetracycline incorporation assay, evaluating mineral apposition rate (MAR, μm/day), and bone formation rate (BFR/B.Pm μm2/μm/day). Three more sections were stained with Stevenel’s Blue and van Gieson Pichrofucsin for histological investigations [48].

Sheep: Each medial and lateral sample was cut in half along the central axis of the implant. One half was not decalcified and was processed for resin embedding; the other half was decalcified and embedded in paraffin. The latter samples were fixed in 10% formalin and decalcified in a nitric-formic acid solution. After decalcification, samples were dehydrated in a graded series of alcohols and then processed for paraffin embedding. Five-micrometer-thick sections were obtained with a Microm HM340E (Microm International GmbH, Germany) and stained with hematoxylin and eosin.

The non-decalcified specimens were first fixed in 4% paraformaldehyde, dehydrated in graded series of alcohols, and then embedded in polymethyl methacrylate (Merck, Schuchardt, Germany). Blocks were sectioned along a plane parallel to the long axis of the osteochondral transplant (EXAKT GmbH & Co., Remscheid, Germany). Sections were then grounded up to a thickness of 40 ± 10 µm (Saphir apparatus 550, ATM GmbH, Germany), stained with Safranin-O/Fast Green, and finally evaluated histologically (BX51, Olympus Optical Co. Europe GmbH, Germany).

The Pineda score was used for semiquantitative evaluation of the specimens [49].

#### 4.3.4. Immunohistochemical Analysis

Sheep: Two sections of decalcified and paraffin-embedded samples were immunostained for col I, col II, and VEGF. After fixation, sections were extensively rinsed in PBS and permeabilized by incubation in 0.3% hydrogen peroxide in PBS solution. Sections to be immunostained were pre-treated for antigen unmasking with 0.2% Pronase (Sigma-Aldrich, St. Louis, MO, USA) solution in PBS. Subsequently, 10% normal serum was added to block nonspecific antibody binding, and primary antibodies (Thermo Fisher Scientific Inc, Waltham, MA, USA) were applied. After rinsing in PBS, slides were incubated with appropriate biotinylated secondary antibody and with horseradish peroxidase-streptavidin complex (Bethyl Laboratories, Inc, Montgomery, TX, USA). Sample reaction was developed with 3,3-diaminobenzidine substrate and permanently mounted.

### 4.4. Statistical Analysis

Statistical analysis was performed using IBM SPSS Statistics v.21. Data are reported as mean ± SD at a significance level of *p <* 0.05. After having verified normal distribution and homogeneity of variance, the non-parametric Kruskal Wallis test, followed by U Mann–Whitney, was used to analyze the data.

## 5. Conclusions

The hybrid MgHA–collagen–chitosan biomaterial used in this study clearly failed to promote bone and osteochondral regeneration in two different animal models. This underlines not only the challenges of regenerating the osteochondral unit, but also the necessity of in-depth in vivo testing before introducing newly developed, although promising, biomaterials into clinical use. Further studies are needed to explore the best formulation of chitosan-reinforced composites for bone and cartilage treatment.

## Figures and Tables

**Table 1:** Macroscopic scores at six months for untreated and experimental groups.

Group	Gross Appearance Score Modified by Fortier et al.		Gross Morphology Score Modified by Niederaurer et al.	
	CFM 15 min < 0 max	CFL 15 min < 0 max	CFM 0 min < 8 max	CFL 0 min < 8 max
Untreated group	5.5	8	3	3
Experimental group	9.5	9.25	3.25	3

**Table 2:** Pineda histology score results. All *p* values > 0.05.

Parameters	Group	N	Mean	Std. Deviation
Defect Filling	Untreated	4	3.00	0.82
	Chitosan	8	1.75	0.89
Osteochondral Junction Reconstruction	Untreated	4	1.00	0.82
	Chitosan	8	0.63	0.52
Matrix Staining	Untreated	4	2.25	1.50
	Chitosan	8	1.25	1.39
Cell Morphology	Untreated	4	2.50	1.29
	Chitosan	8	2.38	0.74
Total	Untreated	4	8.75	3.59
	Chitosan	8	6.00	2.45

## Supplementary Materials

Supplementary Materials can be found at https://www.mdpi.com/1422-0067/20/9/2227/s1.
