# Supplementary material for: A Composite Chitosan-Reinforced Scaffold Fails to Provide Osteochondral Regeneration
Source: Int J Mol Sci. 2019 May 7;20(9):2227. doi: 10.3390/ijms20092227 (PMC6539239; doi:10.3390/ijms20092227)

Supplemental Materials

Materials and Methods

Animal Anesthesia: Details

Rabbit Model:

General anesthesia was induced by intramuscular injection of 44 mg/kg ketamine (Imalgene 1000, Merial Italy S.p.A, Milan, Italy) and 3 mg/kg xylazine (Rompun Bayer SpA, Milan, Italy) under assisted ventilation with O_2/air (1/0.4 l/min) mixture and 2.5% isoflurane (Forane, Abbot SpA, Latina, Italy).

Antibiotics and analgesic therapy after the surgery: A total of 0.6 ml/kg flumequil (Flumexil, FATRO SpA, Bologna, Italy) and 0.1 ml/kg/day metamizole sodium (Farmolisina, Ceva Vetem SpA, Grosseto, Italy).

Sheep Model:

General anesthesia was induced by pre-medication with 10 mg/kg ketamine i.m. (Ketavet 100, Farmaceutici Gellini S.p.A., Latina, Italy), 0.3 mg/kg xylazine i.m. (Rompun Bayer AG, Germany), and 0.0125 mg/kg atropine sulfate s.c.; induction with 6 mg/kg sodium thiopentone i.v. (2.5%); and maintenance with O_2, air, and 2%–3% sevorane (Sevorane, ABBOTT Srl, Latina, Italy) under assisted ventilation (Servo Ventilator 900 D, Siemens, Germany).

During the postoperative period, antibiotic (cephalosporin 1 g per day for 5 days, Cefamezin Pfizer Italia Srl, Latina, Italy) and analgesic (metamizole sodium 50 mg/kg/die for 3 days; Farmolisina Ceva Vetem,S.p.A, Milan, Italy) therapy was administered i.m.

Micro-CT Evaluation: Details

Imaging Acquisition

For the rabbits' explants, each sample was rotated by 180° with a rotation step of 0.4° and an average frame of 2. The nominal resolution was 9 µm. For the sheep explants, each sample was rotated by 180° with a rotation step of 0.4° and an average frame of 3. The nominal resolution was 17.5 µm. The acquired images were later reconstructed by the software NRecon (1.6.8.0) with corrections for alignment beam hardening and ring artefact reduction (Fig. 5).

The microtomographic sections were used to create 3D models of the analyzed object that allowed visualizations of the samples in space. Two specific volumes of interest (VOIs) were defined in each sample. TV_1: Cylindrical VOI with diameter of 6 mm and height of 8 mm or diameter of 7 mm and height 5 mm for rabbits and sheep, respectively (used to evaluate the material and the new bone formation into the defect); TV_2: VOI of the trabecular bone of the condyle in the area around the defect with the same height of TV_1 (used to evaluate peri-implant bone).

Morphological Parameters:

1. Defect BV/TV_1 (%), ratio between the volume of newly formed bone within the bone defect and the total volume of the bone defect;
2. Defect Trabecular Thickness Tb.Th (mm), calculated in model independent way [Hildebrand T et al. Comput Methods Biomech Biomed Engin. 1997;1(1):15–23] over the total volume of the bone defect TV_1;
3. Defect Trabecular Number Tb.N (mm^{-1}), the number of intersections through a trabecular structure per unit length of a random linear path through TV_1;
4. Defect Trabecular Separation Tb.Sp (mm), calculated as the Tb.Th;

5. Peri-implant BV/TV_2 (%), ratio between the volume of trabecular bone around the bone defect and the total volume of interest TV_2;

6. Peri-implant Tb.Th Trabecular Thickness, Tb.N Trabecular Number and Tb.Sp Trabecular Separation calculated as above (in TV_2).

Figure 5. Cross-sectional images of the defined VOIs TV_1 and TV_2 highlighted in red on the sagittal sections of the rabbit's condyle. Cross-sectional images of the defined VOIs TV_1 and TV_2 highlighted in red on the axial sections of the sheep condyle.

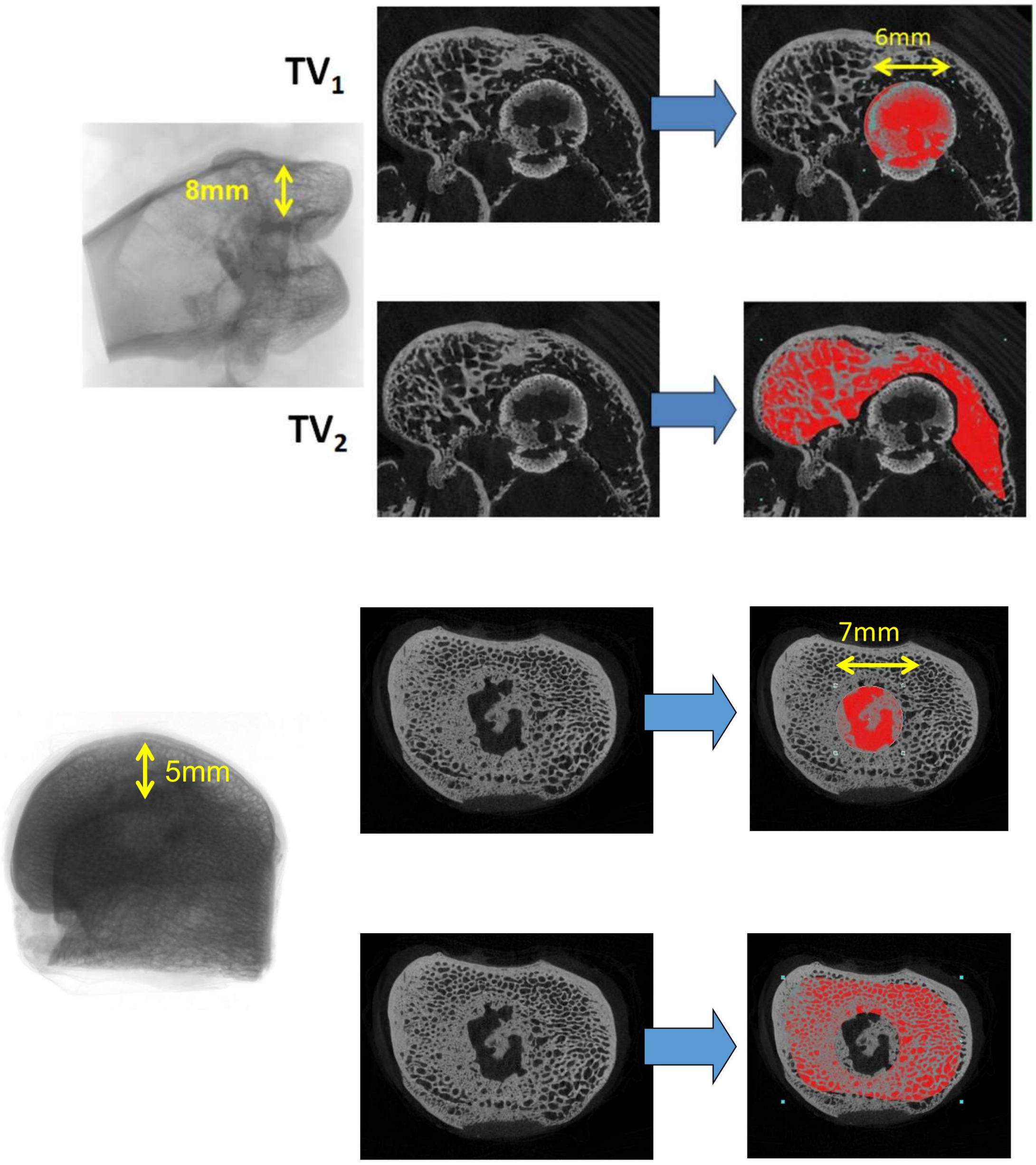

Histological evaluation: details

Evaluation of Oxytetracycline Incorporation:

To assess bone tissue growth through dynamic histomorphometry, the animals received an i.m. injection of oxytetracycline (30 mg/kg) (Terramicina 100, Pfizer Italy, Italy) before the end of the experimental time (two days on, ten days off, two days on).

Oxytetracycline incorporation was evaluated into 5 regions of interest (ROI) near or inside the scaffolds, rating mineral apposition rate (MAR) and bone formation rate (BFR) as previously described

The 5 ROI were grabbed at 20x magnification using a light/fluorescence microscope connected to a digital camera and to an image analysis software (BX51, Olympus Optical Co. Europe GmbH, Germany). Oxytetracycline labelling was evaluated by applying the nomenclature and methodology of the American Society of Bone and Mineral Research (ASBMR) as follows:

1. Mineral Apposition Rate (MAR, µm/day): The distance between the midpoints of two consecutive deposited and epifluorescent fronts of fluorochrome divided by the time between the labelling period.

2. Bone Formation Rate (BFR/B.Pm µm2/µm/day): Obtained by multiplying the MAR value by the sum of half the single label perimeter (sL.Pm) and double the label (dL.Pm) perimeter.

Immunohistochemical Analysis: Details

After fixation, sections were extensively rinsed in PBS and permeabilized by an incubation in 0.3% hydrogen peroxide in PBS solution. Sections to be immunostained were pre-treated for antigen unmasking with 0.2% Pronase (Sigma, Mo, USA) solution in PBS. Subsequently, 10% normal serum was added to block nonspecific antibody binding, and the primary antibodies (Thermo Fisher Scientific Inc, USA) were applied. After rinsing in PBS, slides were incubated with appropriate biotinylated secondary antibody and with horseradish peroxidase–streptavidin complex (Bethyl

Laboratories, Inc, TX). Sample reaction was developed with 3,3-diaminobenzidine substrate and permanently mounted.

RESULTS

Rabbits Microtomography Evaluation

Figure 6: Results of calculated 3D parameters relevant to newly formed bone inside the defect and peri-implant bone inside TV2, on control (group 1) and on experimental group (group 2).

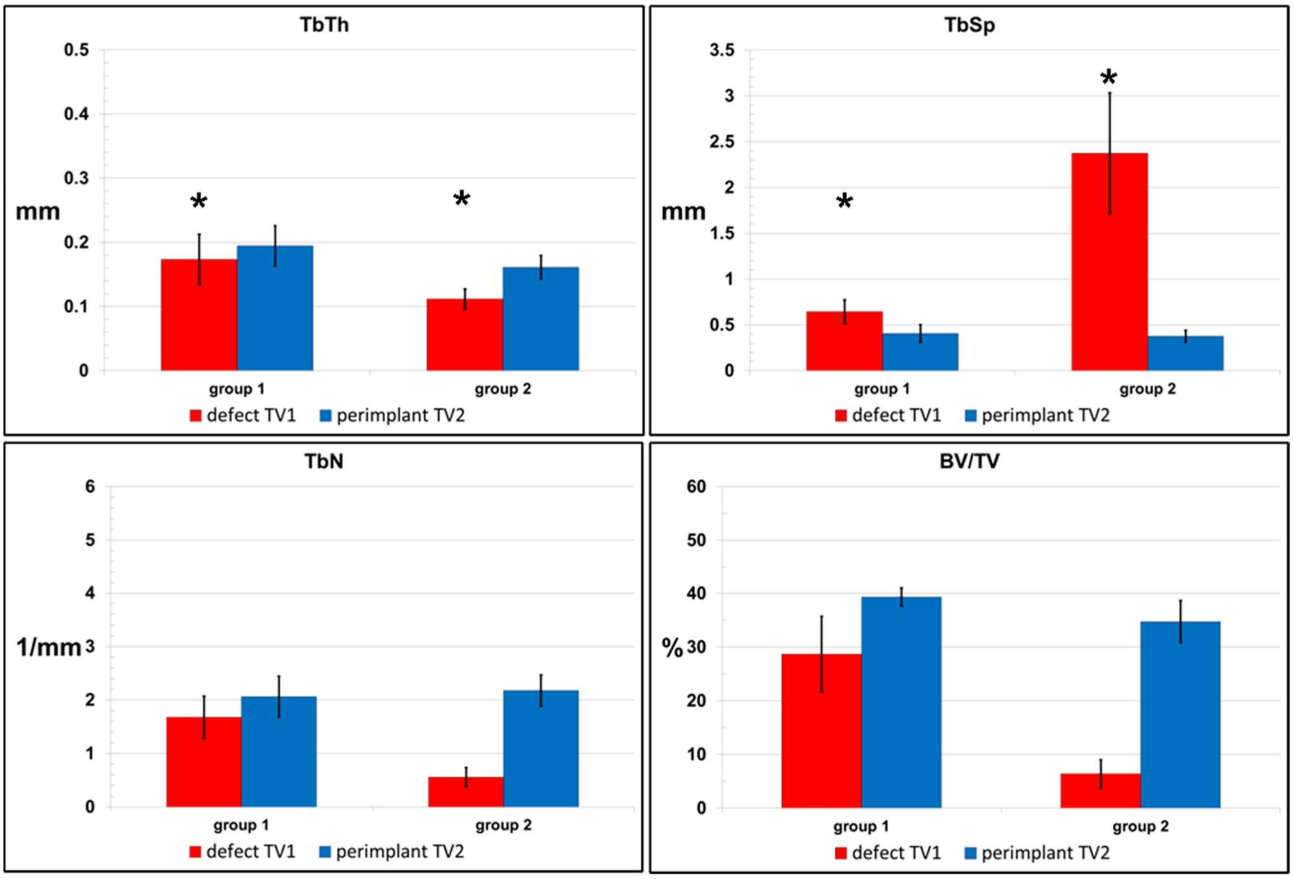

Sheep Microtomography Evaluation

Figure 7: Results of calculated 3D BV/TV, TbTh, TbN, and TbSp parameters relevant to newly formed bone inside the defect and peri-implant bone inside TV2, on control (group 1) and on experimental group (group 2).

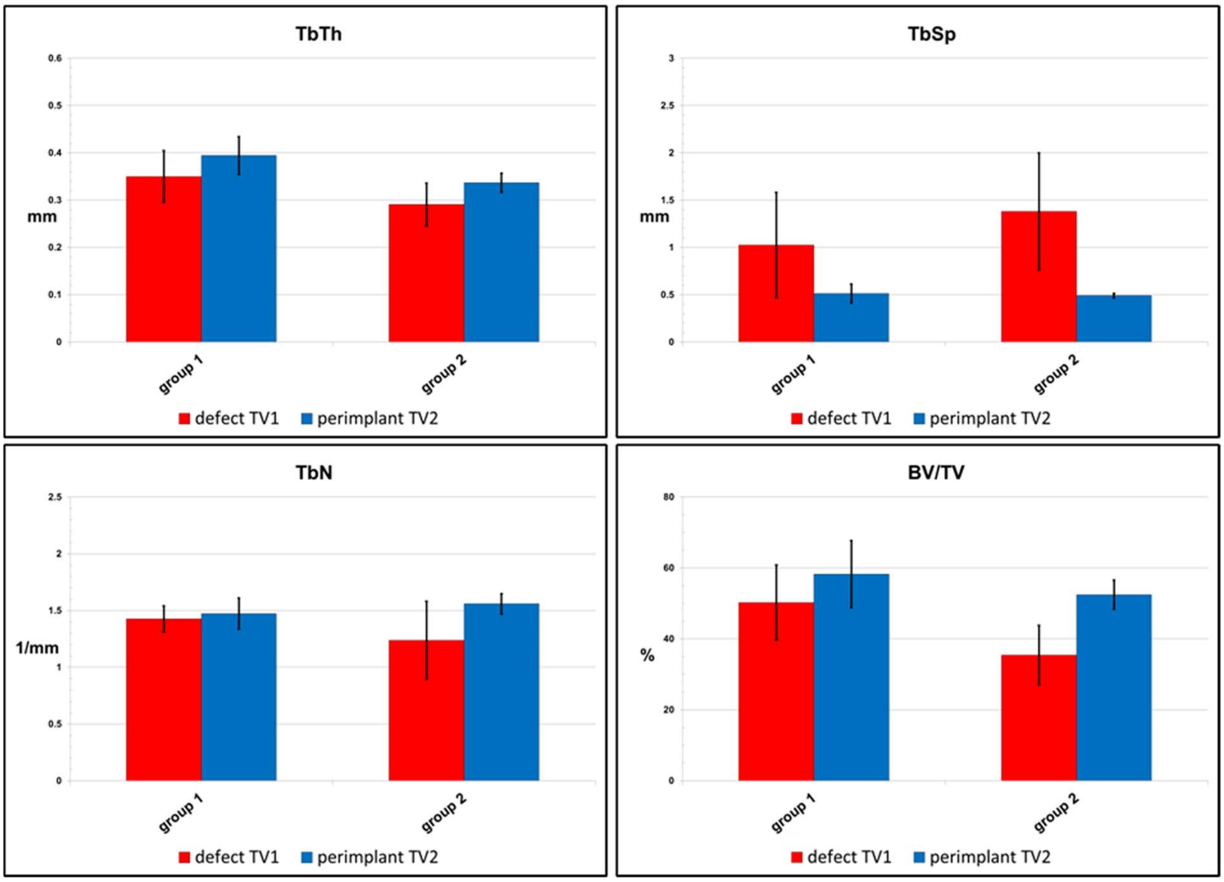

Supplement: Supplementary file 1 [file ijms-20-02227-s001.pdf]
